# Whole genome sequence datasets of *Salmonella enterica* serovar Saintpaul ST50 and serovar Worthington ST592 strains isolated from raw milk in Brazil

**DOI:** 10.1016/j.dib.2023.109965

**Published:** 2024-01-04

**Authors:** Elma L. Leite, Mauro M.S. Saraiva, Priscylla C. Vasconcelos, Daniel F.M. Monte, Marc W. Allard, Patrícia E.N. Givisiez, Wondwossen A. Gebreyes, Oliveiro C. Freitas Neto, Celso J.B. Oliveira

**Affiliations:** a Department of Animal Science, College for Agricultural Sciences, Federal University of Paraiba (CCA/UFPB), Areia, PB, Brazil; b São Paulo State University (Unesp), School of Agricultural and Veterinarian Sciences, Jaboticabal, SP, 14884-900, Brazil; c Office of Regulatory Science, Center for Food Safety and Applied Nutrition, U. S. Food and Drug Administration, College Park, MD, USA; d Department of Preventive Veterinary Medicine, College of Veterinary Medicine, Ohio State University, Columbus, OH, USA; e Department of Preventive Veterinary Medicine, Veterinary School, Federal University of Minas Gerais (UFMG), Belo Horizonte, MG, Brazil; f Global One Health Initiative (GOHi), Ohio State University, Columbus, OH, USA

**Keywords:** Foodborne pathogens, *Salmonella enterica*, Salmonellosis, Whole-genome sequencing, One health, Milk safety

## Abstract

Herein we report the draft genome sequences of *Salmonella enterica* subsp. *enterica* serovars Saintpaul ST50 and Worthington ST592 isolated from raw milk samples in Northeastern Brazil. The 4,696,281 bp *S.* Saintpaul ST50 genome contained 4,628 genes in 33 contigs, while *S.* Worthington ST592 genome was 4,890,415 bp in length, comprising 4,951 genes in 46 contigs. *S.* Worthington ST592 carried a conserved Col(pHAD28) plasmid which contains the antimicrobial resistance determinants *tet(C), acc(6′)-Iaa*, and a nonsynonymous point mutation in ParC (p.T57S). The data could support further evolutionary and epidemiologic studies involving *Salmonella* organisms.

Specifications Table

**Table:** 

Subject	Microbiology
Specific subject area	Microbial genomics
Type of data	Raw reads of sequenced genome assembled and annotated draft genome of the strains *Salmonella enterica* serovar Saintpaul ST50 and Worthington ST592.
How the data were acquired	Illumina MiSeq, Unicycler v.1.0, PATRIC server, *Salmonella in silico* Typing Resource (SISTR) tool, MLST 2.0, SPIFinder 1.0, ResFinder, Comprehensive Antibiotic Resistance Database (CARD), Virulence Factor Database (VFDB), PlasmidFinder, BLAST Ring Image Generator (BRIG) 3.0, and NCBI tools: Isolates Browser - Pathogen Detection and AMRFinderPlus.
Data format	Raw; Analyzed.
Description of data collection	Pure cultures of both *Salmonella enterica* serovar Worthington ST592 and serovar Saintpaul ST50 strains were used for total DNA extraction using a commercial kit (QIAsymphony DSP DNA, Qiagen). Genomic libraries were prepared and sequenced in the MiSeq platform (Illumina). Assembled genomes obtained from raw reads were annotated and analyzed for in silico multilocus sequence typing, antimicrobial resistance genes, stress response genes, virulence factors, identification of *Salmonella* Pathogenicity Islands (SPIs), and plasmids.
Data source location	Institution: LAPOA-Federal University of Paraiba (UFPB). City/Town/Region: Areia, Paraíba. Country: Brazil.
Data accessibility	The datasets are hosted in a public repository. *Salmonella enterica* subsp. *enterica* serovar Saintpaul ST50: Bioproject Accession Number: PRJNA593524. NCBI GenBank Accession Number: JAHPIQ000000000.2. NCBI BioSample Accession Number: SAMN19730533. *Salmonella enterica* subsp. *enterica* serovar Worthington ST592: Bioproject Accession Number: PRJNA593524. NCBI GenBank Accession Number: JAHPIR000000000.2. NCBI BioSample Accession Number: SAMN19730733. Direct URL to data: https://www.ncbi.nlm.nih.gov/nuccore/JAHPIQ000000000 and https://www.ncbi.nlm.nih.gov/nuccore/JAHPIR000000000. Genome annotation information of both strains is available at Science Data Bank repository (DOI: 10.57760/sciencedb.13449) and refer to the Supplementary Materials S1, S2, S3, S4, S5, S6 and S7. Direct URL to data: https://www.scidb.cn/en/detail?dataSetId=87cf4da6599d4d1d8b1f80b5dba6925e&version=V1

## 1 Value of the Data

• *Salmonella enterica* is a leading foodborne pathogen causing salmonellosis, a major zoonosis affecting populations in both developed and developing regions worldwide. While there is a plethora of publicly available sequencing data of *Salmonella enterica* serovars originating from livestock such as pigs and poultry, those associated with milk and dairy products in Brazil are still scarce.
• The present whole genomes sequencing data describe genomic-related features associated with important *Salmonella* serovars in agri-food systems.
• The data herein reported for both isolates can provide valuable information supporting further studies on comparative genomics addressing the epidemiology and evolution of *Salmonella*.

## 2 Objective

The consumption of raw milk or dairy products made with raw milk, such as cheese, has been associated with foodborne illness outbreaks worldwide, highlighting the importance of hygiene practices to mitigate *Salmonella* in the dairy production chain. The recent advances in high throughput sequencing technologies in parallel with ever-lower costs provide the opportunity to obtain in-depth genomic information on critical pathogens to public health. In addition, such information has the potential to trigger important changes toward the implementation of high-resolution monitoring and surveillance systems in the food industry. However, the success to improve monitoring and surveillance systems on a global scale depends on the availability of genomic information. Therefore, this study aimed at providing key genomic features of *S.* Saintpaul ST50 and *S.* Worthington ST592 isolated from contaminated raw milk in Northeastern Brazil.

## 3 Data Description

Here we report the whole genome sequencing data of *Salmonella enterica* subsp. *enterica* serovar Saintpaul ST50 and serovar Worthington ST592 strains, genome screening for antimicrobial resistance (AMR) and virulence factors. Additionally, multilocus sequence typing, identification of *Salmonella* Pathogenicity Islands (SPIs), and plasmids-related data are also described.

The *S.* Saintpaul genome was assigned as sequence type (ST) 50, and comprised 1325,200 reads, with 304,796,000 bases and an average of 215 bases per read. The assembly generated 33 contigs with an N_50_ value of 416,750 bp and 60-fold coverage with a chromosome of 4696,281 bp comprising 4628 coding sequences (CDS), 22 rRNA genes, and 78 tRNA genes. This genome presented 52.2% G + C contents.

**Fig. 1:**
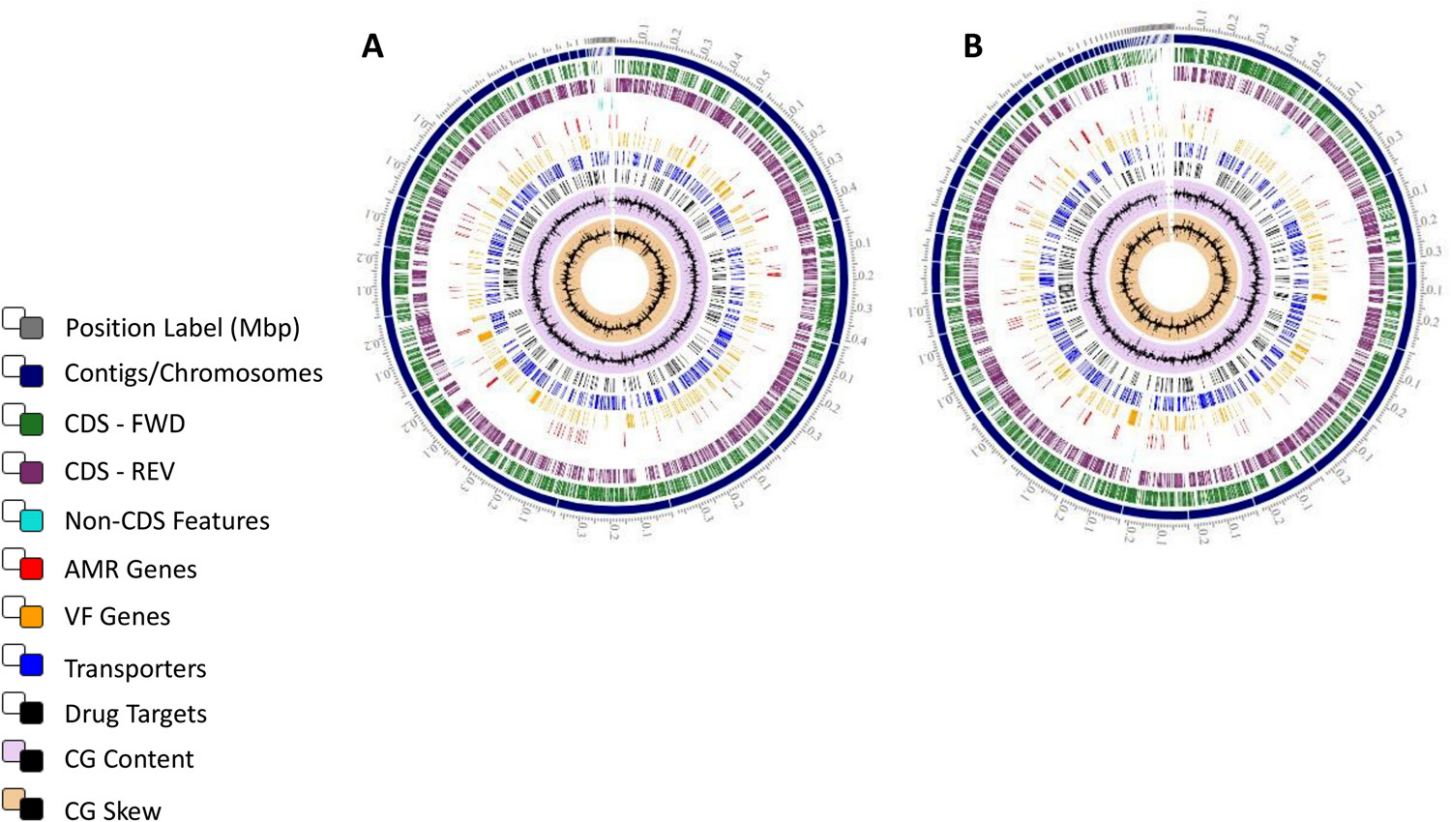
Circular genome maps of *Salmonella* Saintpaul ST50 (A) and *Salmonella* Worthington ST592 (B) constructed by means of the Comprehensive bacterial bioinformatics resource known as PATRIC. From the outer to the inner ring - contigs (scale - x1Mbp), coding sequence (CDS) in the direct strand, CDS in the reverse strand, RNA genes, CDS with homology to known antimicrobial resistance genes, CDS with homology to known virulence factors, GC content, and GC skew.

The *S.* Worthington genome was assigned as ST 592. A total of 1487,452 reads were obtained averaging 294 bases per read with 446,235,600 total bases. 46 contigs were generated after assembly, with an N_50_ value of 238,900 bp and 78X of the coverage, with a G + C content of 52.2%. The chromosome was 4890,415 bp in length, comprising 4951 CDS, 12 rRNA genes, and 79 tRNA genes. A graphical representation of the annotated genomes is shown in (Fig. 1A and B).

According to the disc-diffusion test, the *S.* Worthington strain was phenotypically resistant to ciprofloxacin (quinolone), gentamicin (aminoglycoside), and tetracycline, while *S.* Saintpaul was sensitive to all tested antimicrobials and considered pan-susceptible. Results of the antimicrobial susceptibility test of the two *Salmonella* strains are shown in (Table 1).

**Table 1:** Results of antimicrobial susceptibility testing by disc-diffusion method for both *Salmonella* Saintpaul ST50 and *Salmonella* Worthington ST592 strains.

Antimicrobials	Inhibition Zone Diameters (mm)	
	*Salmonella* Saintpaul	*Salmonella* Worthington
Cephalothin	25	25
Chloramphenicol	27	27
Tetracycline	25	**6**
Ceftriaxone	29	27
Amoxicillin/clavulanic acid	28	26
Ciprofloxacin	22	**6**
Gentamicin	22	**8**
Ceftiofur	25	25
Sulfamethoxazole	21	21
Ampicillin	24	22
Streptomycin	18	19
Kanamycin	22	19

Bolded values indicate resistance.

Downstream analyses showed that both strains harbored genes encoding resistance to aminoglycosides [*aac(6′)*-*Iaa*]. We also provided detailed information on the genes identified in both CARD and VFDB databases as supplementary data (Supplementary materials S1, S2, S3 and S4). The *S.* Worthington ST592 genome harbored one plasmid Col(pHAD28) (KU674895), whilst no plasmids were identified in the *S.* Saintpaul ST50 genome.

*S.* Worthington ST592 genome carried a tetracycline resistance gene [*tet(C)*]. *S.* Worthington ST592 harbored a non-synonymous point mutation in *parC* (p.T57S) associated with the substitution of threonine by serine (Thr/Ser) as detected by both Resfinder and CARD databases (Supplementary material S5), explaining the ciprofloxacin resistance [1]. Resistance mechanisms were not identified using NCBI's Isolate Browser Pathogen Detection resource (https://www.ncbi.nlm.nih.gov/pathogens/isolates).

**Fig. 2:**
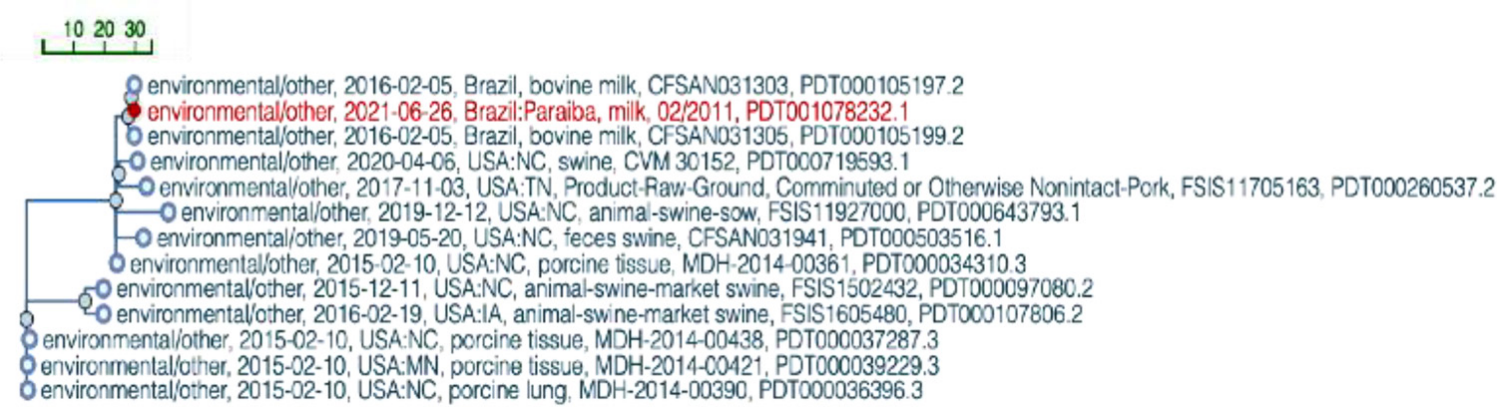
SNP Cluster tree generated by the NCBI's Isolates Browser tool for the *Salmonella* Worthington ST592 genome. The genome sequence of *S*. Worthington ST592 strain isolated from the raw milk in Northeastern Brazil is represented in red.

While *S.* Worthington ST592 was highly related to *S.* Worthington strains originating from swine in the USA (SAMN14504800; SAMN13542975; SAMN03577474; SAMN07968656; SAMN02699527) (Fig. 2), *S.* Saintpaul ST50 strain did not cluster with other lineages deposited at the NBCI database (data not shown).

Additional analyzes performed by NCBI's AMRFinderPlus showed that *S.* Saintpaul ST50 harbored the stress response genes *golT* and *golS,* while *S.* Worthington ST592 harbored *pcoA, pcoB, pcoC, pcoD, pcoE, pcoR, pcoS, silP, silA, silB, silF, silC, silR, silS, silE, golT* and *golS*. Both strains had the virulence genes *iroC* and *iroB* (Table S6 and Table S7- supplementary data).

**Fig. 3:**
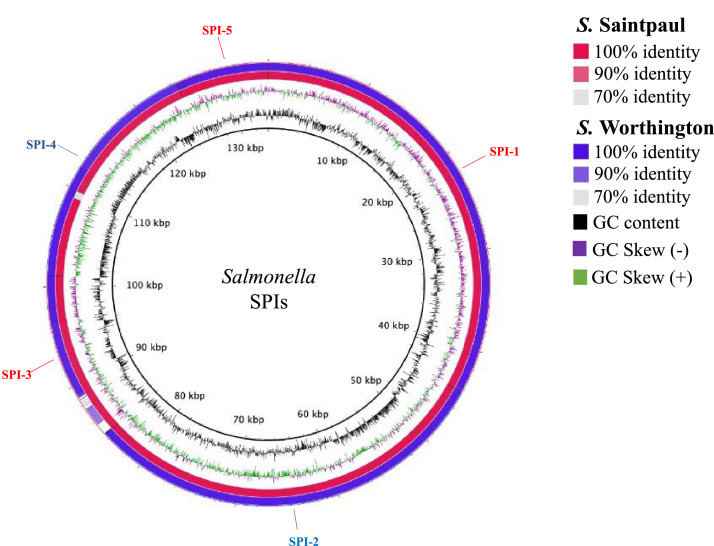
*Salmonella* Saintpaul ST50 and *Salmonella* Worthington ST592 strains constructed by means of the BLAST Ring Image Generator known as BRIG. The red circle represents the genome sequence of the *S.* Saintpaul ST50 strain, and the blue cycle indicates the sequence of the *S.* Worthington ST592 genome. The black circle indicates the GC content and green and purple GC skew. *S.* Typhimurium LT2 was used as the reference genome. The red arrows show positions of the *Salmonella* Pathogenicity Islands (SPI-1, SPI-3, and SPI-5) while the blue arrows depict the positions of the SPI-2 and SPI-4. (For interpretation of the references to color in this figure legend, the reader is referred to the web version of this article.)

The comparative structural analysis of both genomes with *S.* Typhimurium LT2 is shown in Fig. 3. We identified the presence of the five major pathogenic islands: SPI-1 which plays a key role in the process of host cell invasion, SPI-2, 3, and 4 related to bacterial survival and growth, and SPI-5 which appears to mediate inflammation and chloride secretion [2,3].

## 4 Experimental Design, Materials, and Methods

### 4.1 Salmonella isolates

The two *Salmonella enterica* isolates were obtained from a cross-sectional investigation involving 197 randomly collected raw milk samples from 50 herds in Northeastern Brazil [4]. Briefly, sterile stainless-steel ladles were used to collect milk samples (100 mL), which were promptly kept on ice until processed within a 4-hour timeframe. Upon arrival at the laboratory, subsamples (25 mL) were subjected to pre-enrichment in 225 mL lactose broth (LB). Aliquots of 0.1 mL and 1 mL were subsequently transferred to 9.9 mL of Rappaport–Vassiliadis (RV) broth and 9 mL of Muller–Kauffmann Tetrathionate Broth (TT), respectively. The RV tubes were then incubated at 42 °C for 24 h, while the TT tubes were incubated at 37 °C for the same time. Thereafter, loopfuls of the enriched cultures were streaked onto xylose lysine desoxycholate agar (XLD) and Hektoen enteric agar (HE) plates. Following incubation at 37 °C for 24 h, characteristic *Salmonella* colonies were selected and inoculated into triple sugar iron agar (TSI) and lysine iron agar (LIA) slants. Confirmation of *Salmonella* isolates was achieved by slide agglutination test employing polyvalent somatic (anti-O) and flagellar (anti-H) antisera.

### 4.2 Antimicrobial susceptibility testing

Antimicrobial susceptibility test of both *Salmonella enterica* isolates was performed by Kirby Bauer disc-diffusion method using the following antimicrobial drugs: ampicillin 10 µg/mL (AMP), amoxicillin / clavulanic acid 20/10 µg/mL (AMC), ceftiofur 30 µg/mL (CTF), ceftriaxone 30 µg/mL (CRO), cephalothin 30 µg/mL (CEF), chloramphenicol 30 µg/mL (CHL), ciprofloxacin 5 µg/mL (CIP), gentamicin 10 µg/mL (GEN), kanamycin 30 µg/mL (KAN), streptomycin 10 µg/mL (STR), sulfamethoxazole 23.75 µg/mL (SUL) and tetracycline 30 µg/mL (TET). The inhibition zone diameters were evaluated according to CLSI guidelines [5]. *Escherichia coli* ATCC 25,922 was used for quality control purposes.

### 4.3 Extraction of DNA and whole genome sequencing

Total DNA extraction was performed using a commercial kit (QIAsymphony DSP DNA, Qiagen). DNA integrity was visually assessed on 1% agarose gel and quantified by fluorometry (Qubit, LifeTechnologies, Carlsbad, CA, United States). Library preparation and paired-end sequencing was achieved using a 500-cycle (2 × 250) MiSeq V2 kit (Illumina, Carlsbad, CA, USA). Before genome assembly, the quality of raw reads was assessed with the FastQC software (https://www.bioinformatics.babraham.ac.uk/projects/fastqc/). We used Trimmomatic [6] for removing Illumina adaptors and low-quality reads (Phred score <20). The reads were *de novo* assembled using Unicycler [7]. Gene predictions and functional annotations were performed using the PATRIC server [8].

### 4.4 Screening for antimicrobial resistance genes (AMR) and virulence determinants and other downstream analyzes

Multilocus sequence typing (MLST) was determined *in silico* by MLST 2.0 [9]. *Salmonella* Pathogenicity Islands (SPIs) was detected by means of SPIFinder 1.0 [10], while the presence of antimicrobial resistance genes was investigated by ResFinder 4.1 [11], and Comprehensive Antibiotic Resistance Database (CARD) [12]. The prediction of virulence genes was performed through the Virulence Factors of Pathogenic Bacteria (VFDB) platform [13]. The serovar was confirmed using the *Salmonella in silico* Typing Resource (SISTR) tool [14]. Isolates Browser (https://www.ncbi.nlm.nih.gov/pathogens/isolates/), and AMRFinderPlus [15] were also used for further investigation regarding AMR determinants, stress response, and virulence genes.

BLAST Ring Image Generator (BRIG) version 3.0 [16] was used for genome comparisons. The circular genomic map was constructed with BLAST + using standard parameters. *S.* Typhimurium LT2 (GenBank accession number AE006468.2) was used as the reference genome.

### 4.5 Screening for plasmids

Plasmid sequences were predicted by means of PlasmidFinder [17]. The analyses were carried out at the Center for Genomic Epidemiology (CGE) web server using raw reads and a 90% threshold identity.

## Ethics Statements

The work meets the ethical requirements for publication in Data in Brief. The work does not involve studies with animals and humans.

## CRediT authorship contribution statement

**Elma L. Leite:** Writing – original draft, Data curation. **Mauro M.S. Saraiva:** Writing – review & editing, Methodology. **Priscylla C. Vasconcelos:** Methodology. **Daniel F.M. Monte:** Methodology. **Marc W. Allard:** Methodology, Writing – review & editing. **Patrícia E.N. Givisiez:** Writing – review & editing. **Wondwossen A. Gebreyes:** Writing – review & editing. **Oliveiro C. Freitas Neto:** Writing – review & editing. **Celso J.B. Oliveira:** Supervision, Project administration, Writing – review & editing.

## Data Availability

Salmonella enterica subsp. enterica serovar Worthington (Original data) (GenBank). Salmonella enterica subsp. enterica serovar Saintpaul ST50 (Original data) (GenBank). Whole genome sequence datasets of Salmonella enterica serovar Saintpaul ST50 and serovar Worthington ST592 strains isolated from the raw milk in Brazil (Reference data) (Science Data Bank).

## Appendix Supplementary materials

Image, application 1

Image, application 2

Image, application 3

Image, application 4

Image, application 5

Image, application 6

Image, application 7
