# Supplementary material for: Whole genome sequence datasets of Salmonella enterica serovar Saintpaul ST50 and serovar Worthington ST592 strains isolated from raw milk in Brazil
Source: Data Brief. 2024 Jan 4;53:109965. doi: 10.1016/j.dib.2023.109965 (PMC10904156; doi:10.1016/j.dib.2023.109965)
Supplement: Supplementary file 5 [file mmc5.docx]

**Supplementary material S1:**

**Table S1:** Description of *parC* gene detected by *in silico* analysis from *Salmonella enterica* serovar Worthington Sequence Type 592 strain using ResFinder (<https://cge.food.dtu.dk/services/ResFinder/>).


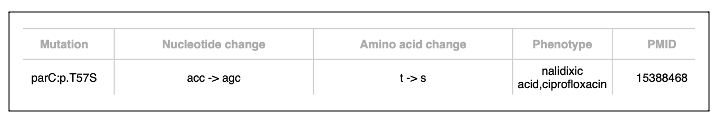


***In silico* identification of the *Salmonella enterica* serovar Worthington Sequence Type 592 strain.**

*In silico* analyses were performed to investigate the gene presence *parC* in the *S.* Worthington ST592 genome. For this, genome assembly was performed employing Unicycler [1] and annotation by the RAST server [2] and later analyzed on the PATRIC genome analysis server (<https://www.patricbrc.org/>) [3]. Figure S1 shows the position of the DNA locus of the (DNA topoisomerase IV subunit A (EC 5.99.1.3) in the genome of *S.* Worthington ST592 strain. We result demonstrated that the *parC* gene is located in the position between 351693-353951 bp with 2259 bp.

**Figure S1:** Shows the gene position for (DNA topoisomerase IV subunit A (EC 5.99.1.3) in the genome of *S.* Worthington ST592 strain. Result extracted from the annotation in RAST [2] by PATRIC genome analysis server (https://www.patricbrc. org/) [3].


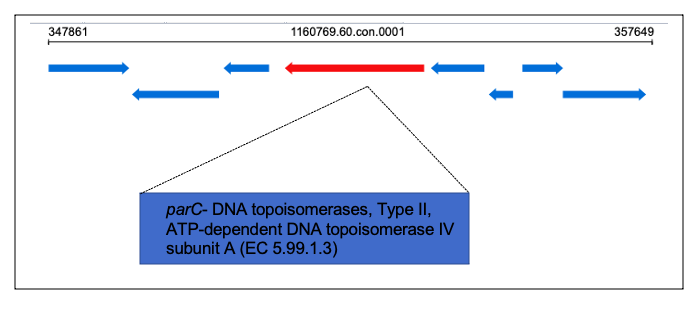


**Nucleotide sequence of the *parC* gene**

The *parC* gene is composed of 2259 bp (position between 351693-353951 bp) in the *S.* Worthington ST592 genome. It was selected through the PATRIC genome analysis server Program (<https://www.patricbrc.org/>) 3. The nucleotide sequence of the *parC* gene is shown below:

>fig|1160769.60.peg.370| DNA topoisomerase IV subunit A (EC 5.99.1.3) [*Salmonella enterica* subsp. *enterica* serovar Worthington *S*_worthington | 1160769.60]

atgagcgatatggcagagcgccttgcgctacatgaatttacggaaaacgcctacttaaac

tactccatgtacgtgatcatggatcgtgcgttgccgtttattggcgacggcctgaagccg

gtacagcgccgcatcgtctatgcgatgtcagagctggggctgaacgccagcgctaaattt

aaaaaatccgcccgtaccgtcggtgacgtactgggtaagtatcatccgcacggcgacagc

gcctgctatgaagccatggtgctgatggcgcagccgttctcttaccgttacccgctggtc

gatggccagggaaactggggcgcgccggatgatccgaagtcattcgcggcaatgcgttat

accgaatctcgcctgtccaaatatgccgagctgctgttaagcgaactcggccaggggacg

gcggactgggtgccaaacttcgacggcacgatgcaggaaccgaaaatgttaccggcgcgt

ctgccgaacatcctgctgaacggcaccaccggtatagcggtggggatggcaacagatatc

ccgccgcacaacctgcgcgaagtggcgaaagcggcgattacgctgattgagcagccgaaa

acgacgctggatcagttgctggatatcgtccaggggccggattacccgaccgaagcggag

atcattacccctcgtgcggaaattcgtaaaatttacgaaaacgggcgtggctccgtgcgt

atgcgcgcggtatggaccaaagaagacggcgccgtggtaatttccgcgctgccgcatcag

gtatctggcgcaaaagtgctggagcagattgctgcgcagatgcgcaataaaaaactgccg

atggtggacgacctgcgcgatgaatcggatcacgaaaacccgacgcgtttagtgattgtg

ccacgctccaaccgtgtggatatggaacaggtgatgaaccatctgttcgccaccaccgat

ctggaaaaaagctaccgtattaacctgaacatgatcggtctggatggtcgtccggcggtg

aaaaacctgctggagatcctcaccgagtggctggcgttccgccgcgacacggtacgccgt

cgtctgaactaccgtctggagaaagtgcttaagcgcctgcatatcctcgaaggtttgctg

gtggcgtttctcaacatcgacgaagtgattgagattatccgtaacgaggatgagccgaaa

cccgcgctgatgtcgcgtttcggcatcagcgaaacccaggcggaagcgattctcgaactg

aaactgcgccatctcgccaaactggaagagatgaaaattcgcggcgagcaggacgagctg

gaaaaagagcgggaccagttgcagggcattctcgcgtccgaacgcaaaatgaataccttg

ctgaaaaaagagctacaggcagattccgacgcctatggcgacgatcgccgttctccgctg

cgtgagcgcgaagaagctaaagcgatgagcgaacacgacatgctgccgtccgaaccggtg

actatcgtgctgtcgcagatgggctgggtgcgcagcgccaaaggtcatgatattgatgcg

ccggggcttaactataaagcgggcgacagctttaaagccgcggtgaaaggtaagagcaat

caaccggtggtgtttattgataccaccgggcgcagctatgctattgatcccattacgctt

ccctcggcgcgtgggcagggcgagccgctgaccggcaaactcacactgccgccgggggcg

accgtagagcatatgctgatggaaggcgatgaccagaaactgctgatggcgtcggatgcg

ggctacggcttcgtttgtacgtttaacgatctggttgcccgtaaccgtgccggtaaggcg

ttgattacactgccggaaaatgcgcacgtcatgccgccgctggtgattgaagacgagcac

gatatgctgctggcgattacccaggccggacggatgttgatgttcccggtagactctctg

ccgcagctgtcgaaaggcaaagggaataagattattaatatcccctctgcggaagcggcg

aaaggcgatgatggactggcgcacctgtacgtgctgccgccacaaagcactctgactatc

catgtcgggaagcgcaaaatcaaactgcgccctgaagagttacagaaggtggtcggcgaa

cgcggacgccgtggcacattaatgcgcggcctgcagcgtatcgatcgcattgagattgat

tcaccgcatcgcgtaagtcatggcgacagcgaagagtaa

**References**

[1] R.R. Wick, L.M. Judd, C.L. Gorrie, K.E. Holt. Unicycler: Resolving bacterial genome assemblies from short and long sequencing reads. PLoS Comput Biol. 13 (2017) e1005595.

[2] R.K. Aziz, D. Bartels, A.A. Best, M. DeJongh, T. Disz, R.A. Edwards, K. Formsma, S. Gerdes, E.M. Glass, M. Kubal, F. Meyer, G.J. Olsen, R. Olson, A.L. Osterman, R.A. Overbeek, L.K. McNeil, D. Paarmann, T. Paczian, B. Parrello, G.D. Pusch, C. Reich, R. Stevens, O. Vassieva, V. Vonstein, A. Wilke, O. Zagnitko. The RAST Server: Rapid Annotations using Subsystems Technology. BMC Genomics. 9 (2008) 75.

[3] A.R. Wattam, J.J. Davis, R. Assaf, S. Boisvert, T. Brettin, C. Bun, N. Conrad, E.M. Dietrich, T. Disz, J.L. Gabbard, S. Gerdes, C.S. Henry, R.W. Kenyon, D. Machi, C. Mao, E.K. Nordberg, G.J. Olsen, D.E. Murphy-Olson, R. Olson, R. Overbeek, B. Parrello, G.D. Pusch, M. Shukla, V. Vonstein, A. Warren, F. Xia, H. Yoo, R.L. Stevens. Improvements to PATRIC, the all-bacterial Bioinformatics Database and Analysis Resource Center. Nucleic Acids Res. 45 (2017) D535–D542.
